# Stent-Graft Relining in a Patient with Acute Aortic Aneurysm and a Completely Migrated Endograft

**DOI:** 10.21470/1678-9741-2017-0122

**Published:** 2017

**Authors:** Jayandiran Pillai, Ceyhan Yazicioglu, Mahad Omar, Martin G. Veller

**Affiliations:** 1 Division of Vascular Surgery and Department of Surgery, University of the Witwatersrand, Johannesburg, South Africa.

**Keywords:** Endovascular Procedures, Aortic Aneurysm, Abdominal, Stents/adverse effects, Blood Vessel Prosthesis/adverse effects, Blood Vessel Prosthesis Implantation/adverse effects, Minimally Invasive Surgical Procedures/adverse effects

## Abstract

Stent-graft migration and type I endoleaks are associated with a higher rate of reintervention and increased mortality and morbidity. This article describes a patient presented with an infrarenal aortic stent-graft which had migrated into the aortic sac with loss of all aortic neck attachment. The acutely expanding abdominal aortic aneurysm was treated by placing a second modular endograft within and above the migrated stentgraft. The patient returned 36 months later, with features of an acute myocardial infarction, severe bilateral lower limb ischemia, and renal failure. He was too ill for intervention and demised within 48 hours.

**Table:** 

Abbreviations, acronyms & symbols	
AAA	= Abdominal aortic aneurysm
CT	= Computed tomography
EVAR	= Endovascular aneurysm repair
MI	= Myocardial infarction
MG	= Migrated stent-graft
SG	= Second modular bifurcated endograft
SVS	= Society for Vascular Surgery

## INTRODUCTION

Endovascular aneurysm repair (EVAR) results in a lower 30-day mortality, compared to open repair^[1,2]^. However, the long term reintervention rate is high^[1,2]^. Migration and type I endoleaks are serious adverse events and indicate EVAR failure^[3,4]^. Distal migration of the main body, within a long infrarenal neck, may be treated by inserting an extender cuff, provided there is some overlap between the main body and neck^[1,2,5]^. More extensive forms of migration, when the main body is no longer contained within the neck (complete migration), may mandate conversion to open repair^[2,3]^. We will describe a medical high-risk patient presented with a completely migrated stent-graft (MG), abdominal pain, and a 10.5 cm infrarenal abdominal aortic aneurysm (AAA). The patient was treated by placing a second modular bifurcated endograft (SG) within and above the MG. Appropriate consent was obtained from the patient.

## CASE REPORT

A 72-year old male presented with abdominal pain. Medical history included hypertension, diabetes and coronary artery disease. He had undergone endovascular repair, 6 cm AAA, five years before and had not returned for follow-up.

**Fig. 1:**
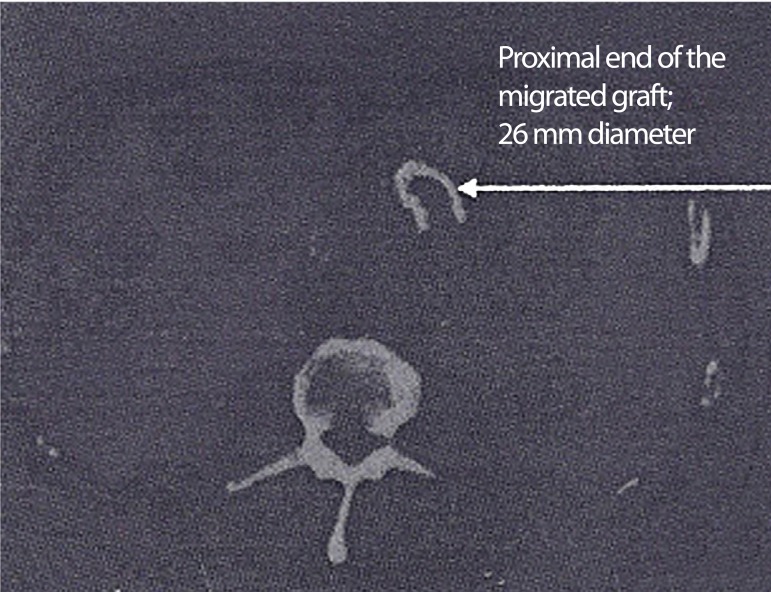
Pre-operative computed tomography (CT) scans: proximal end of migrated graft within the aneurysm sac.

The computed tomography (CT) scan revealed a 10.5 cm infrarenal AAA and a totally migrated AneuRx^TM^ stent-graft (Medtronic Inc. Minneapolis, USA). There was a complete displacement and angulation of the stent-graft's main body within the aneurysm sac (Figure 1). Both iliac components of the endograft were still maintained inside their respective iliac arteries (right limb as part of the main body and left limb as an intact extension). Due to his body mass index and comorbidities, we opted for endovascular stent-graft placement.

### Management Strategy and Planning

The plan was to place a SG within and above the existing MG. The short SG's (Anaconda^TM^ Stent Graft System Vascutek Terumo, Inchinnan Renfrewshire, Glasgow, Scotland) main body should lie within the infrarenal neck and the two iliac limbs to extend itself from the main body into the iliac limbs of MG. All components were oversized by 10%.

### Procedure

The main body of SG (24F) was introduced from the right femoral artery and deployed just below the renal arteries. The distance between the distal end of SG and the proximal end of MG was 40 mm. The left limb of SG's main body was cannulated by attaching the ipsilateral and contralateral magnets within the MG's main body. The contralateral magnet wire was then exchanged for a Lunderquist wire. A 17 mm x 140 mm contralateral iliac limb was used to bridge the gap between SG and MG, landing into the left iliac limb of MG. An ipsilateral 17 mm x 140 mm iliac limb was deployed from the right iliac limb of SG into the right limb of MG. Thus, a new modular endograft was inserted above and into the MG, thereby excluding the aneurysm sac from blood flow. Intraoperative angiogram didn't reveal endoleaks. Pedal pulses were palpable postoperatively. The duration of the procedure was 1 hour and 30 minutes.

**Fig. 2:**
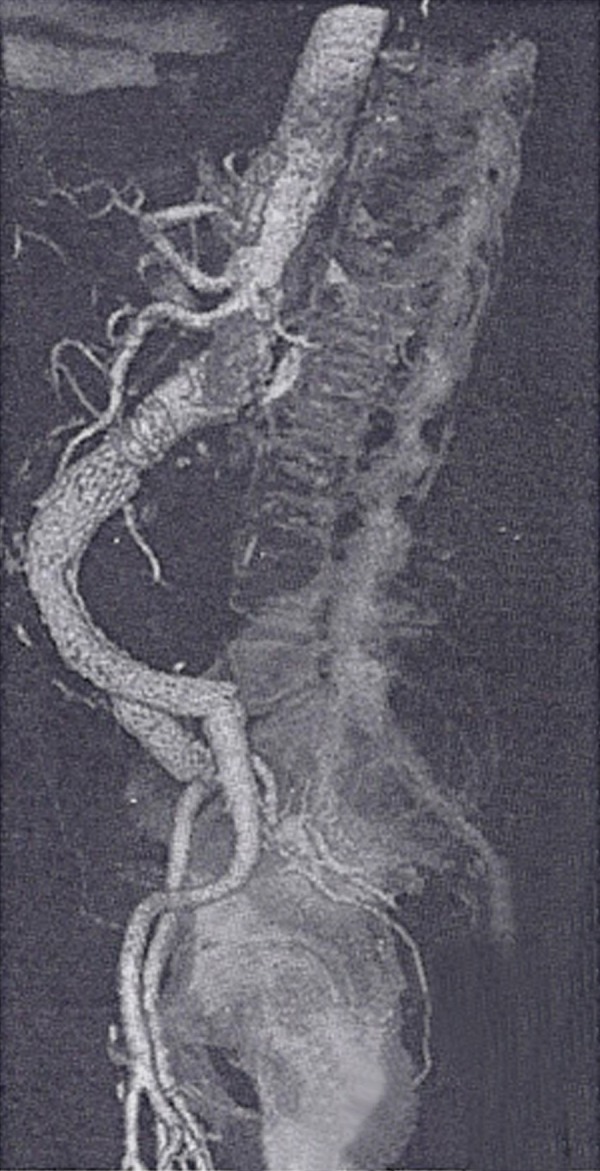
Six weeks follow-up computed tomography (CT) scan.

The patient was discharged 72 hours later. CT scans at 6 weeks, 6 months and 18 months showed intact endograft components, absence of endoleaks and shrinkage of the aneurysm sac (Figure 2). He returned after 36 months with chest pain, hypotension and loss of sensation in both lower limbs. Investigations confirmed acute myocardial infarction (MI), lower limb ischemia and severe renal dysfunction. Ultrasound revealed absence of blood flow within the stent-graft. He was assessed as being too ill for any cardiological or vascular intervention. He demised within 48 hours of admission. It was unclear whether it was the lower limb ischemia or cardiac ischemia that initiated his current condition.

## DISCUSSION

The Society for Vascular Surgery (SVS) define migration as the movement of the endograft by 10 mm relative to anatomical landmarks or any migration leading to symptoms or requiring therapy^[6]^. There are no guidelines for treating complete migration^[5]^. Open repair is the recommended procedure of choice to treat complete migration.

In this case, due to inflammation, graft component incorporation and the prohibitive comorbidities, endovascular repair seemed to be a better option. The use of an extender cuff only was not an option because the graft had completely migrated. The Anaconda^TM^ device was chosen. Its short main body allowed a "working length" to place two iliac extension limbs between SG and MG. The lower limb ischemia prior to his death could have been due to component occlusion, kinking or migration. Stent-graft thrombus secondary to MI and hypotension was also possible. In appropriate individuals, such "off-label" use of EVAR devices requires a stricter follow-up or even conversion to open repair at a later stage if the patient's general condition allows it.

Although EVAR relining by using a second infrarenal endograft seems to be a feasible alternative to immediate open surgery in high-risk patients, with a complete MG, other options need to be investigated. Customizing EVAR devices in this setting demands more investigation.

**Table:** 

Authors' roles & responsibilities	
JP	Substantial contributions to the conception or design of the work; final approval of the version to be published
CY	Substantial contributions to the conception or design of the work; final approval of the version to be published
MO	Substantial contributions to the conception or design of the work; final approval of the version to be published
MGV	Substantial contributions to the conception or design of the work; final approval of the version to be published
